# The burden of childhood cancers in the South Asian Association for Regional Cooperation (SAARC) Region: a population-based, cross-sectional GLOBOCAN 2022 analysis

**DOI:** 10.1016/j.lansea.2026.100815

**Published:** 2026-07-14

**Authors:** Tara Pattilachan Menon, Jazlyn Selvasingh, Taral K. Jella, C.S. Pramesh, Sanjeeva Gunasekera, Venkatraman Radhakrishnan, Maya Prasad, Swati Goel, Shakeel Modak, Suzanne L. Wolden, Christopher B. Jackson, Bishal Gyawali, Edward Christopher Dee

**Affiliations:** a Virginia Tech Carilion School of Medicine, Roanoke, VA, USA; b National Cancer Grid and Department of Surgical Oncology, Tata Memorial Hospital, Tata Memorial Centre, Homi Bhabha National Institute, Mumbai, MH, India; c National Cancer Institute, Maharagama, Sri Lanka; d Department of Medical Oncology, Pediatric Oncology Division, Cancer Institute Adyar, Chennai, India; e Division of Pediatric Oncology, Tata Memorial Centre, Mumbai, Maharashtra, India; f Department of Internal Medicine, University of California San Francisco School of Medicine, San Francisco, CA, USA; g Department of Pediatrics, Memorial Sloan Kettering Cancer Center, New York, NY, USA; h Department of Radiation Oncology, Memorial Sloan Kettering Cancer Center, New York, NY, USA; i Division of Cancer Care and Epidemiology, Cancer Research Institute, Queen's University, Kingston, ON, Canada; j Departments of Oncology and Public Health Sciences, Queen's University, Kingston, ON, Canada

**Keywords:** Childhood cancer, Pediatric oncology, Cancer epidemiology, Cancer burden, South Asia, GLOBOCAN

## Abstract

**Background:**

Countries of the South Asian Association for Regional Cooperation (SAARC) are home to approximately 600 million children aged 0–14 years, representing a quarter of the global child population. We report incidence and mortality for childhood cancers among South Asia's eight nations to inform priority setting for research, programming for health services, and policy for childhood cancers.

**Methods:**

Incidence and mortality of new childhood cancer diagnoses for all cancers combined and for selected leading cancer types were retrieved from GLOBOCAN 2022 database of Afghanistan, Bangladesh, Bhutan, India, Maldives, Nepal, Pakistan, and Sri Lanka. Age-standardized incidence rates (ASR) and mortality-to-incidence ratios (MIR) were calculated, together with statistical examination of cross-country variation in diagnostic structure and mortality patterns.

**Findings:**

In SAARC nations, 37,716 new childhood cancer diagnoses and 17,698 deaths were estimated in 2022. India contributed 68.8% of subregional cases (25,939), and Pakistan contributed 20.8% (7841). Age-standardized incidence rates differed and were greatest in Sri Lanka (10.4/100,000) and Pakistan (10.1) and lowest in Bhutan (2.3) and Bangladesh (3.7). Age-standardized mortality rates ranged from 1.1/100,000 in Bhutan to 5.2/100,000 in Afghanistan (SAARC-wide ASMR 3.5/100,000). Boys represented 57% of cases overall, with the proportion varying across countries. Leukemia was the most common cancer in all countries (35–50%), followed by central nervous system (CNS) tumors (approximately 12% of cases). MIR ranged from 0.30 (Sri Lanka) to 0.57 (Afghanistan).

**Interpretation:**

SAARC childhood cancer patterns reflect both population size and health system capacity, with a preponderance of leukemia and marked mortality-to-incidence ratio disparities across countries, especially for CNS cancers. There is a need to have subregional cooperation to address gaps in cancer registration, equitable access to specialty care, and continuity of treatment.

**Funding:**

ECD is funded in part through the 10.13039/100000892Prostate Cancer Foundation Young Investigator Award. SM, SLW, CBJ, and ECD are funded in part through the Cancer Center Support Grant from the 10.13039/100000054National Cancer Institute (P30 CA008748).

**Research in context**

**Evidence before this study**

Prior international and regional analyses have compared adult and childhood cancer burden across world regions, and single-country studies have characterized childhood cancer incidence, mortality, and care-seeking patterns within individual SAARC nations. However, no published analysis has systematically compared childhood cancer incidence, mortality, sex-specific patterns, and cancer-type composition across all eight SAARC countries using a single, internationally standardized data source, leaving regional policymakers without a comprehensive, comparable evidence base for priority-setting.

**Added value of this study**

Using GLOBOCAN 2022 estimates, this study provides a comprehensive, cross-country comparison of childhood cancer incidence, mortality, sex-specific differentials, and cancer-type composition across all eight SAARC nations. We report region-wide male:female age-standardized rate ratios for both incidence (1.39) and mortality (1.42), identify substantial heterogeneity in mortality-to-incidence ratios (0.30 in Sri Lanka to 0.57 in Afghanistan) that point to disparities in case ascertainment and care access rather than survival alone, and characterize country-specific cancer-type heterogeneity using standardized residual analysis.

**Implications of all the available evidence**

These findings highlight wide intraregional disparities in childhood cancer burden and outcomes that are not explained by population size alone, and they underscore the need for expanded population-based cancer registration, especially in Afghanistan, Bangladesh, Bhutan, the Maldives, and Nepal, alongside coordinated subregional investment in pediatric oncology infrastructure and survivorship care, to close measurable gaps in childhood cancer control across South Asia.

## Introduction

The South Asian Association for Regional Cooperation (SAARC) region, encompassing Afghanistan, Bangladesh, Bhutan, India, Maldives, Nepal, Pakistan, and Sri Lanka, represents one of the world's most populous areas, containing over 2 billion people—more than a quarter of the global population.^1^ Within this demographic landscape, children aged 0–14 years constitute approximately 600 million individuals, making SAARC home to the largest childhood population among world regions.^2^ Despite this massive demographic footprint, the region faces profound healthcare challenges, with health expenditure averaging only 4% of GDP compared to 10% globally, and severe shortages of specialized medical personnel.^3^

Childhood cancers, while individually rare, collectively represent a critical health challenge across SAARC nations.^4^^,^^5^ Compared to adult malignancies, childhood cancers are highly curable, yet their outcomes are heavily influenced by healthcare system capacity, diagnostic infrastructure, and socioeconomic determinants.^6^^,^^7^ The burden of childhood cancer extends beyond immediate medical care to encompass long-term survivorship needs, family financial toxicity, and health system resource allocation in settings where competing priorities for maternal and child health services are abundant.^7^^,^^8^

The epidemiological landscape of childhood cancer across SAARC countries exhibits considerable heterogeneity, reflecting complex interactions between genetic predisposition, environmental exposures, healthcare accessibility, and cancer registration completeness.^9^, ^10^, ^11^ Countries like Sri Lanka have invested substantially in cancer control infrastructure, including national registries and standardized treatment protocols, while others face significant challenges in basic diagnostic capacity and specialist workforce development.^12^, ^13^, ^14^, ^15^ These disparities are compounded by cultural factors influencing healthcare-seeking behaviors, gender-based differences in care access, and the prevalence of traditional medicine practices that may delay formal medical consultation.^16^, ^17^, ^18^, ^19^, ^20^, ^21^, ^22^, ^23^

International cancer burden analyses have compared global regions for both adult and childhood populations.^24^^,^^25^ Prior studies have examined the patterns and determinants of general cancer burden across SAARC nations and of childhood cancer in single-country studies.^26^, ^27^, ^28^ However, less is known specific to childhood cancer in SAARC nations. Significant healthcare system developments, population demographic shifts, improved registry coverage, and refined estimation methodologies have collectively altered the childhood cancer landscape, necessitating an updated analysis.

This study seeks to characterize and compare the magnitude, distribution, and mortality patterns of childhood cancers across all eight SAARC countries using GLOBOCAN 2022 estimates. We examine cross-country heterogeneity in incidence rates, cancer profile, sex-specific patterns, and mortality patterns to identify regional commonalities and country-specific challenges. In the context of healthcare system diversity and projected demographic changes, we discuss implications for cancer control policy, resource allocation, and regional collaboration frameworks to improve outcomes for children with cancer across South Asia.

## Methods

Incident cases and deaths from childhood cancers for all cancers combined and selected major cancer types were collected for the eight South Asian Association for Regional Cooperation (SAARC) countries—Afghanistan, Bangladesh, Bhutan, India, Maldives, Nepal, Pakistan, and Sri Lanka—representing 2022 populations among children aged 0–14 years. GLOBOCAN 2022 is produced by the International Agency for Research on Cancer (IARC) and combines country-level registry data on cancers with vital statistics as well as region-based modeling where registry coverage has been partial or absent. More details about GLOBOCAN data methodology and hierarchy of registries utilized are available in Supplementary Table S1. This study used publicly available, aggregate, de-identified country-level data and was deemed exempt from institutional review board review; no individual informed consent was required.

Cancer types were classified using standard GLOBOCAN site group labels (for example, “Leukemia” and “Brain, central nervous system”) aligned with the ICD-O–3 groupings used in the estimation pipeline. For each country and sex, the total number of estimated childhood cancer cases was taken from the ‘All cancers' row. Residual catch-all categories (for example, “Other specified”) were retained and considered in interpreting the overall site mix. Cancer incidence and mortality estimates were analyzed for both sexes combined, and separately by sex where data were available. Sex was recorded in GLOBOCAN as a binary variable (male/female) based on registry records; gender identity data were not available in these population-level estimates, precluding gender-based analyses. GLOBOCAN does not provide individual-level information on race, ethnicity, caste, or other sociocultural identity markers for childhood cancers in these countries, so we could not examine within-country inequities by race or ethnicity.

Age-standardized incidence rates (ASIRs) and age-standardized mortality rates (ASMRs) per 100,000 person-years were estimated using direct standardization to the 1966 Segi–Doll World standard population.^29^ Mortality-to-incidence ratios (MIR) were determined as the ratio of nation-level estimated death counts to estimated incident case counts per 2022 GLOBOCAN figures. We note that MIRs derived from counts rather than age-standardized rates represent a study limitation; findings should be interpreted with this in mind. We retained MIR as the primary mortality indicator because it requires only count data that are available for every SAARC country, and it remains a widely used, accepted metric for population-level cross-country comparison of childhood cancer burden in settings lacking age-stratified mortality counts or robust vital registration. Because MIR is also sensitive to differential case ascertainment and is not itself a survival measure, we interpret cross-country MIR differences as indicative of relative case-ascertainment and health-system disparities rather than as precise estimates of case fatality, and we report MIR alongside, rather than in place of, directly age-standardized incidence and mortality rates wherever age-stratified counts permit. Site-mix analysis characterized each country's cancer profile by calculating the proportional contribution of each cancer type to the total estimated childhood cancer burden, restricted to cancer types with sufficient case counts. For sex-specific patterns, male:female ratios were calculated for sex-specific ASR for all cancers and for selected site-specific categories.

### Statistical analysis

**Fig. 1:**
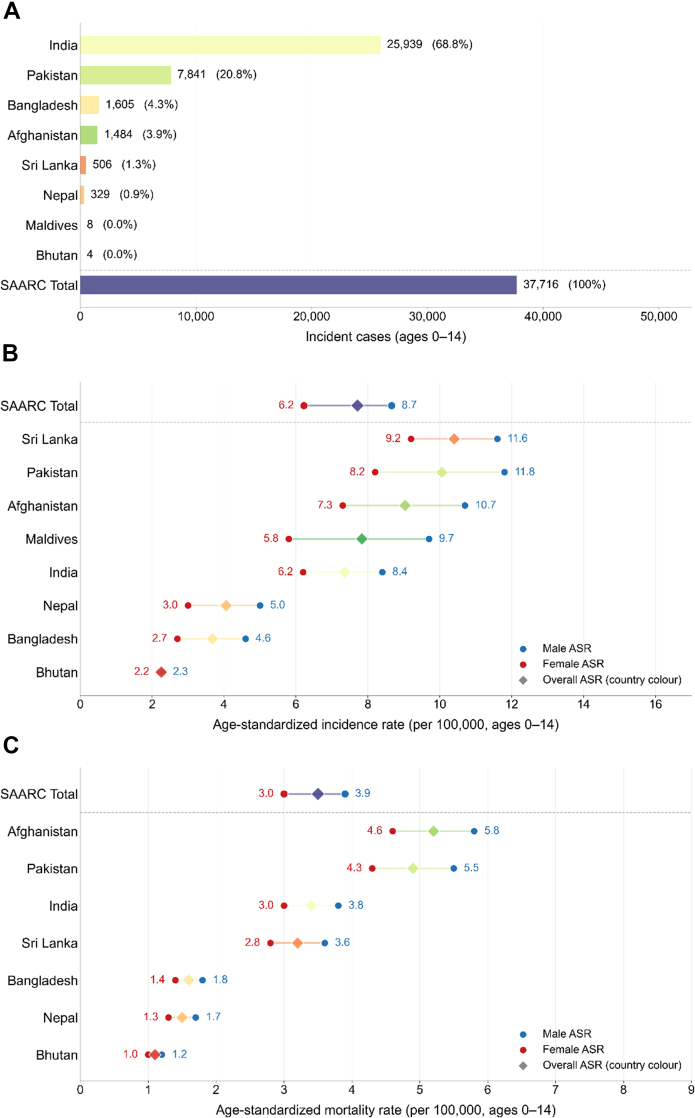
**Childhood cancer incidence in South Asia, 2022 (ages 0–14)**. (A) Absolute number of incident cases by country, with the SAARC regional total shown below the dashed line as a reference benchmark. (B) Age-standardized incidence rates (ASR, per 100,000) by country and sex, ordered by composite ASR from highest (top) to lowest (bottom). Each row shows the female ASR (red circle) and male ASR (blue circle) connected by a line whose length represents the magnitude of the sex gap; the diamond indicates the overall composite ASR, colored by country. (C) Age-standardized mortality rates (ASMR, per 100,000) by country and sex, using the same plot format as panel B but ordered by composite ASMR from highest (top) to lowest (bottom). Each row shows the female ASMR (red circle) and male ASMR (blue circle); the diamond indicates the overall composite ASMR, colored by country. The Maldives is omitted from panel C because mortality data were not available. In both panels B and C, the SAARC total is shown above the dashed line as a reference benchmark.

**Fig. 2:**
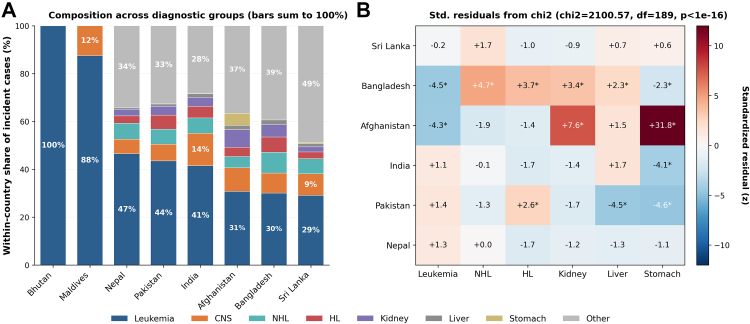
**Cancer profile patterns in childhood cancer (0–14), South Asia (2022).** (A) Stacked bar chart showing the proportional composition of cancer types within each country, with bars summing to 100%. (B) Heatmap of standardized residuals from the chi-square analysis, showing deviations from expected frequencies for six key childhood malignancies across the six countries with sufficient case counts. Colors indicate the direction and magnitude of deviation: red indicates excess cases; blue indicates deficit. Standardized residuals (z-scores) are calculated as (observed − expected)/√expected; asterisks denote statistically significant deviations (|z| ≥ 2). The omnibus chi-square test spans all eight countries and 28 cancer types (χ2 = 2100.57, df = 189, p < 0.001); panel B displays the filtered subset used for residual interpretation.

Statistical tests adhered to best practices for categorical contingency tables and count data with two-sided hypothesis testing at α = 0.05. Pearson's chi-square testing for sex distribution of childhood cancer utilized country × sex contingency tables for all cancers pooled together; no continuity correction occurred for tables more than 2 × 2. Cross-country variation for site composition testing utilized Pearson's chi-square on the country × site matrix after filtering for instability (<5 cases for exclusion). Comparisons among pairwise site-mixes among countries selected for testing utilized constrained intersection sets of high-volume sites with Pearson's chi-square testing. Standardized residuals with an absolute value ≥2 were considered significant contributors and reported descriptively as z-scores. For mortality comparisons, MIRs were calculated from national deaths with incidence to be tested for heterogeneity with a 2 × k table frame (deaths vs non-deaths). Comparisons among regions were performed to determine if particular sites were disproportionate contributors to mortality compared to incidence utilizing Pearson's chi-square testing on aggregated site × outcome tables. Because GLOBOCAN does not provide standard errors for age-standardized rates (ASR [World]), we did not compute confidence intervals for ASR (World) nor perform between-country hypothesis tests on these rates. All inferential analyses were restricted to contingency-table comparisons of counts using Pearson χ^2^ tests. These analytic choices follow established precedent for cancer burden analyses using GLOBOCAN data. As noted in the Data Sources section, all chi-square tests and standardized residuals should be interpreted as descriptive indicators of heterogeneity in the modeled data rather than as formal inferential tests of epidemiological hypotheses. We highlight several further limitations of this approach. This study was not designed to test a priori hypotheses, so all p-values reported here summarize the degree of heterogeneity present rather than confirm or refute a pre-specified clinical or biological hypothesis. Because GLOBOCAN incidence and mortality figures are themselves modeled estimates rather than fully observed counts in several SAARC countries, some of the cross-country and cross-sex heterogeneity captured by these tests may partly reflect the underlying estimation methodology rather than true population differences. Finally, given the large sample sizes involved, even small and clinically unimportant differences can reach statistical significance; readers should therefore weigh the magnitude of the differences reported (for example, in Tables 1 and 2 and Fig 1. and Fig. 2) rather than relying on statistical significance alone to judge their importance.

**Table 1:** Childhood cancer incidence and mortality by country, SAARC region, age 0–14 years, 2022.

	Incident cases	ASR (per 100,000)	Deaths	Mortality rate (per 100,000)	MIR
Afghanistan	1484	9.04	841	5.2	0.567
Bangladesh	1605	3.68	709	1.6	0.442
Bhutan	4	2.25	2	1.1	–
India	25,939	7.36	12,028	3.4	0.464
Maldives	8	7.83	–	–	–
Nepal	329	4.06	121	1.5	0.368
Pakistan	7841	10.06	3843	4.9	0.490
Sri Lanka	506	10.40	154	3.2	0.304
SAARC total	37,716	7.48	17,698	3.5	0.469

Data are n or n (%). – indicates data not available or insufficient case numbers for reliable MIR calculation. ASR = age-standardized rate per 100,000 population.

**Table 2:** Leading cancer types by incidence and mortality, SAARC region, age 0–14 years, 2022.

	Incident cases	Male n (%)	Female n (%)	% of total	Deaths	Male n (%)	Female n (%)	% of total	MIR
Leukemia	15,412	9686 (62.8)	5726 (37.2)	40.9	7215	4545 (63.0)	2670 (37.0)	40.8	0.468
Brain/CNS	4424	2614 (59.1)	1810 (40.9)	11.7	2435	1431 (58.8)	1004 (41.2)	13.8	0.550
Non-Hodgkin lymphoma	2433	1725 (70.9)	708 (29.1)	6.5	1163	824 (70.9)	339 (29.1)	6.6	0.478
Hodgkin lymphoma	1872	1427 (76.2)	445 (23.8)	5.0	865	663 (76.6)	202 (23.4)	4.9	0.462
Kidney	1494	779 (52.1)	715 (47.9)	4.0	612	322 (52.6)	290 (47.4)	3.5	0.410
Ovary	727	0 (0.0)	727 (100.0)	1.9	272	0 (0.0)	272 (100.0)	1.5	0.374
Liver	554	332 (59.9)	222 (40.1)	1.5	274	163 (59.5)	111 (40.5)	1.5	0.495
Other specified cancers	7235	4081 (56.4)	3154 (43.6)	19.2	3299	1873 (56.8)	1426 (43.2)	18.6	0.456
All other cancers	3565	2139 (60.0)	1426 (40.0)	9.5	1563	905 (57.9)	658 (42.1)	8.8	0.438
Total	37,716	21,460 (f)	16,256 (43.1)	100.0	17,698	10,123 (57.2)	7575 (42.8)	100.0	0.469

Data are n or %. Cancer types are ordered by incident case frequency. MIR = mortality-to-incidence ratio. CNS = central nervous system.

### Ethics approval

The Ethical Committee of Memorial Sloan Kettering Cancer Center waived the need for ethical approval or informed consent for this study as the use of publicly available population-level data does not represent human subjects research.

### Role of the funding source

The funders had no role in study design, data collection, data analysis, data interpretation, or writing of this manuscript.

## Results

Total child population (ages 0–14 years) within SAARC nations were an estimated 600 million in 2022, ranging from a low of 89,000 children for Bhutan to a high of over 380 million for India. For South Asia as a region, there were an estimated 37,716 incident cancers among children 0–14 with 17,698 deaths in 2022. Absolute burden remained highly concentrated among the most populated countries: India contributed an estimated 25,939 cases (68.8% of the region's total), Pakistan an estimated 7841 cases (20.8%), Bangladesh an estimated 1605 cases (4.3%), and Afghanistan an estimated 1484 cases (3.9%); Sri Lanka contributed an estimated 506 cases (1.3%), Nepal an estimated 329 cases (0.9%), and Maldives and Bhutan together accounting for an estimated 12 cases (Fig. 1A, Table 1).

Age-standardized incidence rates were heterogeneous throughout the region, with distinctly different patterns from absolute distributions of estimated cases (Table 1, Fig. 1B). High childhood cancer ASR was found for Sri Lanka with 10.40 per 100,000 population, Pakistan (10.06), and Afghanistan (9.04) (Table 1, Fig. 1B). Childhood cancer ASR was 7.36 for India, despite having a very high absolute burden and was 7.83 per 100,000 for the Maldives, while lower rates were observed with Nepal at 4.06, Bangladesh at 3.68, and Bhutan at 2.25 per 100,000 population (Table 1, Fig. 1B).

Age-standardized mortality rates (ASMR) showed a broadly similar but not identical rank order, with notable exceptions; Sri Lanka ranked highest in incidence but fourth in mortality (Table 1, Fig. 1C). ASMRs ranged from 1.1/100,000 in Bhutan to 5.2/100,000 in Afghanistan, with a SAARC-wide overall ASMR of 3.5/100,000. Afghanistan and Pakistan had the highest ASMRs (5.2 and 4.9, respectively), while Bangladesh and Nepal recorded lower rates (1.6 and 1.5). Male ASMRs consistently exceeded female ASMRs across all countries (Fig. 1C). The region-wide male:female ASMR ratio was 1.42 (male ASMR 4.07 vs female ASMR 2.86 per 100,000), slightly exceeding the corresponding region-wide male vs female ASIR ratio of 1.39, indicating that the male excess in mortality modestly outpaces the male excess in incidence.

Males accounted for about 56.9% of new childhood cancers region-wide, with a highly variable male-female distribution by country (χ^2^ = 17.984, df = 7, p = 0.012) (Table 2, Fig. 1B). The region-wide male:female ASIR ratio was 1.39 (male ASIR 8.66 vs female ASIR 6.22 per 100,000). Male-to-female ASIR ratio was higher in Bangladesh (1.71), Maldives (1.66), and Nepal (1.65), intermediate in Afghanistan (1.46), Pakistan (1.44), and India (1.36), lower in Sri Lanka (1.27) (Fig. 1B). Bhutan showed near-equality with 0.97 ratio (Fig. 1B).

Leukemia was the predominant childhood cancer across all SAARC countries, typically comprising 35–50% of national childhood cancers, with Bhutan and Maldives being notable exceptions where leukemia comprised nearly all classified cases (88-100%), reflecting sparse underlying data. (Fig. 2A). However, an omnibus analysis of cancer profile composition revealed profound cross-country heterogeneity (χ^2^ = 2100.57, df = 189, p < 0.001; these analyses should be interpreted as descriptive indicators of heterogeneity given the modeled nature of underlying estimates, as noted in the Methods) and pairwise comparisons showed that India's cancer profile distribution differed markedly from that of Pakistan (χ^2^ = 305.87, p < 0.001) and Bangladesh (χ^2^ = 116.53, p < 0.001). Standard residual analysis pinpointed significant contributors to these patterns: Afghanistan had excess stomach cancer and kidney tumors (z ≈ +31.8 and + 7.6, respectively), India had lower stomach cancer representation (z ≈ −4.1), and Pakistan higher Hodgkin lymphoma (z ≈ +2.6) (Fig. 2B). Brain and central nervous system tumors were the second most frequent cancer regionally, with India having a proportionally higher CNS tumors share compared to neighboring nations (Fig. 2A).

**Fig. 3:**
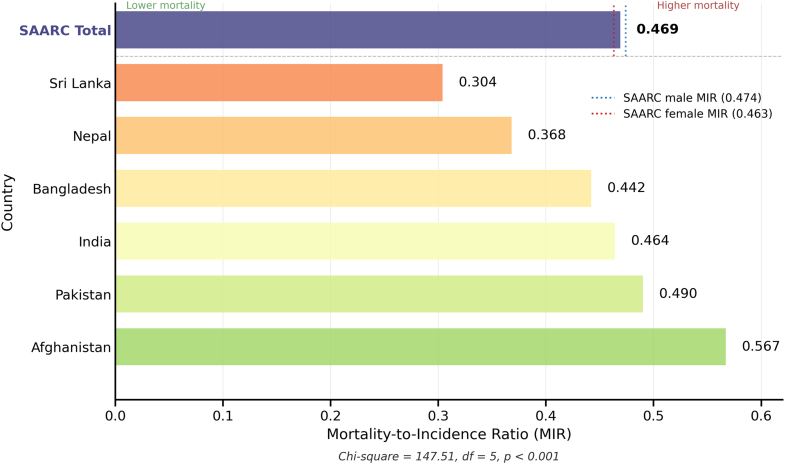
**Childhood cancer mortality-to-incidence ratios across SAARC countries.** Bars represent the overall MIR for each country, calculated as the ratio of cancer deaths to incident cases. Bhutan and the Maldives are omitted because of insufficient case counts for reliable MIR calculation. The SAARC regional total (MIR = 0.469) is shown above the dashed line as a benchmark. Dotted vertical lines within the SAARC Total row indicate the SAARC-wide male MIR (0.474, blue) and female MIR (0.463, red).

The overall MIR in the SAARC region was 0.469 (Table 1). Country-specific MIRs varied substantially across the SAARC region (Table 1, Fig. 3). MIRs ranged from 0.304 in Sri Lanka to 0.567 in Afghanistan, with intermediate values for Pakistan, India, Bangladesh, and Nepal (Table 1, Fig. 3). Cross-country heterogeneity testing confirmed significantly varying mortality proportions (χ^2^ = 147.51, df = 5, p < 0.001) (Table 1, Fig. 3).

**Fig. 4:**
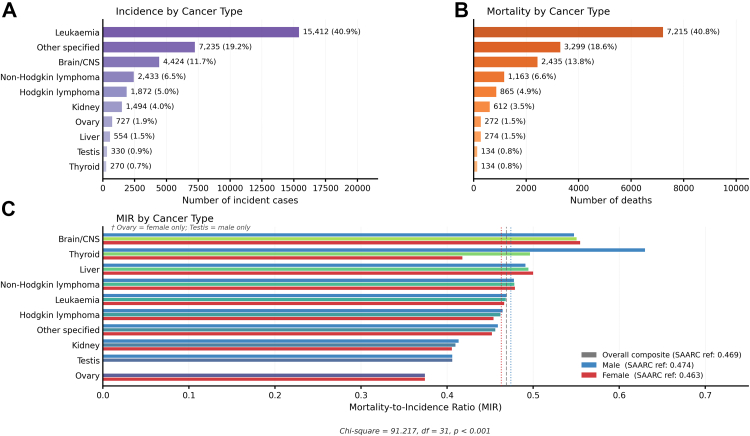
**Childhood cancer incidence, mortality, and MIR by cancer type in the SAARC region.** (A) Absolute number of incident cases by cancer type, with the percentage of the SAARC-wide total shown in parentheses. (B) Absolute number of deaths by cancer type, with the percentage of the SAARC-wide total shown in parentheses. (C) Sex-stratified mortality-to-incidence ratio (MIR) by cancer type. For each cancer type, three bars are shown: overall composite MIR (colored by cancer type), male MIR (blue), and female MIR (red). Vertical reference lines indicate the SAARC-wide overall MIR (dashed gray, 0.469), male MIR (dotted blue, 0.474), and female MIR (dotted red, 0.463). Ovary is female-only and testis is male-only.

Leukemia accounted for 40.8% of all estimated childhood cancer deaths in the SAARC region (approximately 7215 deaths) and 40.9% of all estimated incident cases (Table 2, Fig. 4A). Brain and CNS tumors comprised 11.7% of estimated incident cases and 13.8% of estimated deaths (approximately 2435 deaths) (Table 2). The absolute mortality burden was highly concentrated in the region's most populous nations: India accounted for the largest share of estimated childhood cancer deaths (12,028 deaths, 67.9% of the regional total), followed by Pakistan (3843 deaths, 21.7%), Afghanistan (841 deaths, 4.8%), and Bangladesh (709 deaths, 4.0%) (Table 1). Sri Lanka and Nepal together contributed approximately 275 deaths (0.9% and 0.7% respectively), with Bhutan contributing 2 deaths; mortality estimates were unavailable for the Maldives (Table 1).

The proportion of leukemia deaths varied significantly across countries (χ^2^ = 155.997, df = 5, p < 0.001; Table 3, Fig. 4B), with highest proportions in Pakistan and Nepal and lower proportions in Afghanistan, Sri Lanka, and Bangladesh. Brain and CNS tumor deaths also varied significantly by country (χ^2^ = 134.921, df = 5, p < 0.001; Table 3, Fig. 4B). Disease-specific MIRs ranged from 0.41 (kidney) to 0.55 (CNS tumors), with CNS tumors at 0.55, non-Hodgkin lymphoma at 0.48, and leukemia at 0.47 (Table 2, Fig. 4C). Comparative analysis of SAARC-wide incidence and mortality distributions showed a significant difference between death patterns and cancer profiles (χ^2^ = 91.217, df = 31, p < 0.001; Fig. 4C).

**Table 3:** Country-specific distribution of leading cancer types by mortality, SAARC region, age 0–14 years, 2022.

	Afghanistan	Bangladesh	India	Nepal	Pakistan	Sri Lanka
	n (%)	n (%)	n (%)	n (%)	n (%)	n (%)
Leukemia	254 (30.2)	202 (28.5)	4955 (41.2)	62 (51.2)	1696 (44.1)	46 (29.9)
Brain/CNS	92 (10.9)	69 (9.7)	1946 (16.2)	7 (5.8)	303 (7.9)	18 (11.7)
Non-Hodgkin lymphoma	36 (4.3)	61 (8.6)	811 (6.7)	11 (9.1)	235 (6.1)	9 (5.8)
Hodgkin lymphoma	28 (3.3)	41 (5.8)	593 (4.9)	3 (2.5)	197 (5.1)	3 (1.9)
Kidney	56 (6.7)	37 (5.2)	373 (3.1)	4 (3.3)	140 (3.6)	2 (1.3)
Stomach	72 (8.6)	0 (0.0)	21 (0.2)	0 (0.0)	4 (0.1)	1 (0.6)
All other cancers	303 (36.0)	299 (42.2)	3329 (27.7)	34 (28.1)	1268 (32.9)	75 (48.7)
Total deaths	841 (100.0)	709 (100.0)	12,028 (100.0)	121 (100.0)	3843 (100.0)	154 (100.0)

Data are n (%). Percentages calculated as proportion of total deaths in each country. Countries shown exclude Bhutan and Maldives due to insufficient case numbers. CNS = central nervous system. SAARC = South Asian Association for Regional Cooperation.

## Discussion

The eight SAARC nations collectively face a substantial challenge with childhood cancers, with nearly 38,000 estimated incident cases and over 17,000 estimated deaths annually representing approximately 15% of the global childhood cancer burden; for context, the region comprises 25% of the world's pediatric population.^30^, ^31^, ^32^ This apparent paradox reflects complex interactions between true epidemiological differences, healthcare system capacity for case detection (likely underdiagnosis), and cancer registration completeness rather than simply lower inherent cancer risk. A concurrent four-part Lancet Oncology Series on cancer care across SAARC countries provides important context, documenting that the region's overall cancer mortality-to-incidence ratio substantially exceeds the global average, with region-wide deficits in cancer detection, treatment infrastructure, and oncology workforce—including severely limited specialized pediatric oncology services in Afghanistan, Bhutan, and the Maldives—that directly inform the childhood cancer mortality patterns documented here.^33^, ^34^, ^35^

The absolute burden in India, which accounts for over two-thirds of regional cases (for context, India is home to 69% of the region's childhood population), highlights both population size effects and the critical importance of strengthening childhood oncology infrastructure in the subcontinent's most populous nation. Notably, prior analyses suggest GLOBOCAN may underestimate India's true childhood cancer burden; Arora et al. estimated 52,366 new pediatric cancer cases annually by applying registry-based incidence rates to India's childhood population--roughly double the recent GLOBOCAN estimates--attributing the discrepancy to incomplete case-finding and lower reported incidence rates.^36^ This discrepancy highlights a distinction worth clarifying: the International Incidence of Childhood Cancer (IICC) is a compilation of population-based cancer-registry incidence data, separate from the International Classification of Childhood Cancer, 3rd edition (ICCC-3), the diagnostic classification scheme used to group tumors by site and histology (discussed further in the Limitations). Compilations such as the IICC depend on population-based cancer registries, which, per Supplementary Table S1, exist for only three of the eight SAARC countries analyzed here (India, Pakistan, and Sri Lanka); Afghanistan, Bangladesh, Bhutan, the Maldives, and Nepal have no major national registry of their own. GLOBOCAN 2022, in contrast, provides modeled national-level estimates for all eight SAARC countries, enabling the comprehensive cross-country comparisons central to this analysis, albeit with greater reliance on extrapolation for registry-sparse countries (see Limitations). When comparing SAARC rates with international norms, most nations fell below the global childhood cancer rate of approximately 8.5 per 100,000, with only Afghanistan, Pakistan, and Sri Lanka exceeding this benchmark (Fig. 1B). The higher age-standardized rates observed in Sri Lanka and Pakistan may suggest strong diagnostic access and/or cancer registration or genuine epidemiological differences that merit further investigation. Sri Lanka's investment in comprehensive cancer registration and standardized care pathways likely contributes to both higher reported incidence and more favorable survival outcomes.^12^

The universal predominance of leukemia across SAARC countries aligns with global childhood cancer epidemiology, where acute lymphoblastic leukemia (ALL) typically represents the most common childhood malignancy.^37^ However, the significant cross-country heterogeneity in overall cancer profile suggests important differences in either true disease patterns or diagnostic capabilities. Importantly, some of the observed heterogeneity may also reflect differences in the source registries and reference populations used for GLOBOCAN model-based imputation, particularly in countries lacking population-based cancer registries. In such settings, diagnostic proportions may partially mirror the cancer profiles of neighboring countries that serve as input sources for modeled estimates rather than representing fully independent national epidemiological patterns.

Afghanistan's apparent excess of kidney tumors may parallel patterns observed in other resource-limited settings such as sub-Saharan Africa, where Wilms tumor is often a dominant diagnosis because it presents as a palpable abdominal mass and can be diagnosed clinically without advanced imaging or pathology.^38^ In contrast, leukemias and CNS tumors, which often require laboratory confirmation or neuroimaging, are frequently under-reported in settings with limited diagnostic infrastructure.^39^

The prominence of brain and central nervous system tumors, particularly in India, carries important implications for regional cancer control planning. Management of CNS malignancies require specialized diagnostic imaging and pathology, neurosurgical and neuro-oncology expertise, pediatric radiotherapy capabilities, and intensive supportive care, resources that are severely constrained across much of the SAARC region. The disproportionately high MIR for CNS tumors (0.55 vs 0.47 for leukemia) is consistent with more limited capacity to diagnose and treat these malignancies, though this pattern may also reflect greater underdiagnosis of CNS tumors relative to leukemia in settings with limited neuroimaging.^40^

The consistent male predominance in childhood cancer incidence across SAARC, with significant cross-country variation in magnitude, reveals important intersections between biological factors and social determinants of health. While some male excess is expected based on inherent susceptibility differences (and indeed male predominance in childhood cancer is a consistent pattern observed globally across all world regions), the magnitude of variation across SAARC countries warrants consideration. The extreme ratios observed in Bangladesh (1.71) and Nepal (1.65) may partly reflect social factors such as differential healthcare-seeking, though cross-country differences in modeled incidence estimates may also contribute to apparent variation in sex ratios, and any social or cultural interpretation should be made cautiously, given the descriptive and modeled nature of these data. These patterns suggest that cultural factors and healthcare-seeking behaviors may systematically disadvantage girls in accessing cancer diagnosis and care.^7^^,^^22^^,^^23^^,^^41^ To further evaluate whether such disadvantages extend to mortality outcomes, we examined SAARC-wide male and female MIRs. The male MIR (0.474) marginally exceeded the female MIR (0.463) at the regional level (Fig. 3), which does not directly confirm excess female mortality. However, this comparison is difficult to interpret in settings where girls may be systematically underdiagnosed: if female incidence is undercounted, both the numerator and denominator of the female MIR are affected, potentially masking true outcome disparities. The directly age-standardized male:female mortality ratio reported above (ASMR ratio 1.42, male ASMR 4.07 vs female ASMR 2.86 per 100,000) does not share this artifact, because each sex's ASMR is standardized independently of the other sex's incidence; we therefore regard it as the more directly interpretable measure of the sex differential in mortality burden, and report the male:female MIR comparison alongside it only as a secondary, descriptive indicator.

These patterns align with broader evidence of gender disparities in child health across South Asia, where cultural preferences, economic constraints, and decision-making hierarchies can delay or preclude medical care for girls. A large multi-centre analysis of over 34,000 children from population-based and hospital-based registries in India documented male-to-female incidence rate ratios of 1.69 in Delhi and 1.37 in Chennai, with an even higher male-to-female ratio (2.06) among children seeking treatment at cancer centers.^23^ Notably, the sex disparity was more pronounced in northern India, in private centers compared with subsidized treatment facilities, among patients traveling more than 100 km for care, and in those undergoing hematopoietic stem cell transplantation (male-to-female ratio 2.81), situations demanding greater financial commitment. These findings are consistent with societal factors compounding financial barriers to girls' access to childhood cancer care, though we acknowledge that modeled estimates and registry completeness differences across countries may also contribute to observed sex ratio variation. The more equitable distribution observed in Sri Lanka (1.27) and near-parity in Bhutan (0.97) may reflect different cultural contexts, healthcare system accessibility, or more systematic case-finding approaches that capture cases regardless of sex.

The dramatic variation in MIR across SAARC countries (from 0.304 in Sri Lanka to 0.567 in Afghanistan) is consistent with substantial disparities in childhood cancer outcomes, while recognizing that MIRs are simultaneously shaped by incidence underestimation, incomplete death registration, and model-based incidence derivation in settings with limited registry coverage. Accordingly, MIRs here should be interpreted as composite indicators reflecting both outcome differences and data system capacity rather than as direct measures of survival. The substantial cross-country variation in mortality proportions (χ^2^ = 147.51, df = 5, p < 0.001) and in MIR itself likely reflects differential outcomes arising from a combination of survival differences, incidence underestimation, and registration completeness rather than any single factor, and is consistent with, though not directly attributable to, differences in treatment access, care quality, and support capacity. Sri Lanka's relatively favorable MIR, however, approaches levels seen in upper-middle-income countries and may reflect sustained investment in cancer control infrastructure, free healthcare provision, and systematic treatment protocols.

Conversely, Afghanistan's high MIR reflects the intersection of ongoing conflict, healthcare system destruction, limited specialist capacity, and economic constraints that render advanced cancer care largely inaccessible. The intermediate patterns observed in Pakistan (0.490) and India (0.464) suggest mixed progress, with pockets of excellence alongside persistent barriers to equitable care access.

The elevated MIR rates across much of SAARC cannot be understood without considering treatment abandonment, which represents a critical bridge between epidemiological patterns and lived experience. Published cohort studies from the region document abandonment rates of 16–30% for childhood ALL with financial constraints, geographic barriers, and family education levels serving as primary predictors.^42^, ^43^, ^44^

In Pakistan, single-center analyses identify maternal education, travel distance, and economic hardship as key determinants of treatment abandonment, with reported abandonment rates of 17–22% despite availability of financially subsidized care in some settings.^42^^,^^45^ Similar patterns emerge from Bangladesh, where econometric work documents 16% abandonment rates primarily driven by treatment costs exceeding family financial capacity.^42^ These findings help explain the elevated MIRs observed regionally and underscore why even highly curable diseases like childhood leukemia carry substantial mortality burdens in resource-constrained settings. However, studies have shown that with holistic patient and family support initiatives, rates of incomplete treatment can come down drastically.^46^

The epidemiological patterns documented across SAARC point toward specific, actionable interventions that could substantially improve childhood cancer outcomes through regional collaboration. First, systematic monitoring of sex-disaggregated cancer statistics should guide targeted interventions where sex disparities are widest, potentially including community education, financial support for girls' healthcare, and family engagement strategies.

Second, the disproportionate CNS tumor mortality burden indicates an urgent need for regional capacity building in pediatric neuro-oncology. Collaborative training programs, shared treatment protocols, telemedicine consultation networks, and referral pathway standardization could leverage expertise from regional centers of excellence while building distributed capacity across countries. The high level of expertise and infrastructure required for treating CNS tumors indicates the need for centralization of care.

Third, the success of India's Indian Collaborative Study Group for Childhood Leukemia (ICiCLe) program and the National Cancer Grid framework offer concrete models for SAARC-wide replication.^47^, ^48^, ^49^, ^50^ Standardized treatment protocols, risk-adapted therapy, supportive care guidelines, and systematic outcome monitoring have demonstrated measurable survival improvements in Indian cohorts and could be adapted across diverse healthcare contexts.^51^, ^52^, ^53^ Temporal patterns in specific childhood cancers such as neuroblastoma have been examined in neighboring Asian countries, including analyses of cross-country inequalities in China.^54^ Similar pattern analyses across SAARC would be valuable for tracking progress in cancer control and identifying emerging epidemiological shifts.

Global efforts such as the WHO's Global Initiative for Childhood Cancer (GICC), which aims to achieve at least 60% childhood cancer survival by 2030, provide an important framework for the region.^55^ Directly relevant SAARC initiatives include the South-East Asia Childhood Cancer Network (SEAR-CCN), the Global Platform for Access to Childhood Cancer Medicines (GPACCM)—which supports Bangladesh, Nepal, Sri Lanka, and Pakistan—and the Indian Childhood Cancer Initiative (ICCI) operating across 10 taskforces.^56^^,^^57^ The Initiative's index cancers, including ALL, Burkitt lymphoma, Hodgkin lymphoma, retinoblastoma, Wilms tumor, and low-grade glioma, are particularly relevant in the SAARC region.

The survival disparities documented across SAARC underscore fundamental healthcare system weaknesses that extend beyond cancer-specific interventions. Countries with high MIRs typically lack basic diagnostic imaging, pathology services, reliable medication supply chains, and trained childhood oncology personnel. Addressing these deficits requires sustained investment in healthcare infrastructure, workforce development, and health financing mechanisms that protect families from financial catastrophe.

Regional collaboration through frameworks such as the SAARC itself, combined with international partnerships including WHO initiatives and global cancer control organizations, could accelerate progress through knowledge sharing, joint training programs, bulk medication procurement, and coordinated advocacy for increased health sector investment.

Interpretation of these findings must consider important limitations inherent in GLOBOCAN estimation methodology, particularly for countries lacking robust cancer registration systems. National estimates for Afghanistan, Bangladesh, and Nepal rely substantially on extrapolation from neighboring countries' registry data, potentially obscuring true epidemiological patterns and introducing uncertainty into cross-country comparisons. Afghanistan specifically lacks a population-based cancer registry; GLOBOCAN estimates are derived from modeled rates based on data from its neighboring countries: Tajikistan, Pakistan, and Uzbekistan. The unusually high proportion of stomach cancer observed in Afghanistan likely reflects instability in these modeled estimates for countries with limited primary data rather than a true epidemiological signal, particularly because stomach cancer is notably prominent in Tajikistan's overall mortality profile and does not appear in the top 10 cancers for the other seven SAARC countries.

Bhutan and the Maldives present a particular data limitation: their childhood cancer profiles consist almost exclusively of leukemia, with near-absent representation of other cancer profiles seen across all other SAARC countries.^58^ This pattern almost certainly reflects data generation constraints, including extremely small case counts, reliance on extrapolated rates from neighboring registries, and limited in-country diagnostic infrastructure, rather than a genuine epidemiological signal. These countries are retained in descriptive analyses but interpreted conservatively throughout. Furthermore, GLOBOCAN also does not include information on race, ethnicity, caste, tribe, or indigeneity, limiting our ability to examine within-country inequities along sociocultural lines or to disentangle the roles of structural racism and other forms of marginalization in shaping cancer burden.

Since GLOBOCAN was the primary data source, cancer type breakdown was according to anatomical site only. However, childhood cancer epidemiological studies prefer disease classifications that include histology as seen in the International Classification of Childhood Cancer-3 (ICCC-3) system.^59^ The lack of ICCC-3 classification limits the ability to report incidence by subtype (e.g., ALL vs AML for leukemia, Hodgkin vs non-Hodgkin for lymphoma), and stage-wise distribution is also unavailable. GLOBOCAN includes only malignant neoplasms (ICD-O behavior code /3); therefore benign CNS tumors, which IARC recommends including in childhood CNS estimates, are likely excluded.^60^

The absence of standard errors for age-standardized rates limits formal statistical testing of incidence differences, while our reliance on MIR as a descriptive mortality indicator cannot substitute for comprehensive cohort-based survival analysis.^61^ Moreover, because GLOBOCAN estimates are derived from registry data and modeling, all calculated p-values in our analyses should be interpreted as descriptive indicators of heterogeneity, not as definitive causal contrasts. Future research priorities should include establishing dedicated population-based childhood cancer registries (PBCCRs), as general cancer registries do not capture high-resolution pediatric-specific data such as Toronto staging, survival outcomes, or non-stage prognosticators.^62^

Additional priorities include conducting population-based survival studies, investigating genetic and environmental determinants of cross-country cancer differences, evaluating the effectiveness of targeted interventions to reduce treatment abandonment and improve care equity, and tracking longitudinal trends in childhood cancer burden using internally consistent population-based registry data, noting that direct comparisons across GLOBOCAN cycles are not methodologically appropriate given changes in estimation methods and country coverage across iterations.

In conclusion, the SAARC childhood cancer landscape couples substantial absolute burden concentrated in India with the highest age-standardized risk in Sri Lanka and Pakistan, demonstrates concerning sex disparities that vary significantly across countries, and exhibits profound mortality-to-incidence disparities, most pronounced for CNS tumors. These patterns point toward concrete interventions: systematic equity monitoring with targeted support for girls, CNS-focused capacity building initiatives, strengthened registries to improve case ascertainment, and expansion of standardized leukemia care protocols with comprehensive supportive services. If implemented through coordinated regional collaboration, these evidence-based approaches directly address the largest and most remediable survival gaps documented across South Asia's childhood cancer burden.

## Contributors

TPM and ECD conceived and designed the study. TPM, JS, and TKJ curated the data and did the statistical analyses. CSP, SG, VR, MP, SG, SM, SLW, CBJ, BG, and ECD contributed to the interpretation of the findings and critical revision of the manuscript for important intellectual content. TPM drafted the initial manuscript. All authors reviewed and approved the final version of the manuscript. TPM and JS accessed and verified the underlying data.

## Data sharing statement

All data used in this analysis are publicly available from the International Agency for Research on Cancer's GLOBOCAN 2022 database (https://gco.iarc.who.int). The country- and sex-specific GLOBOCAN 2022 data extracts underlying all reported estimates, together with the analytic code, are provided as a supplementary data file accompanying this submission and are also available from the corresponding author on reasonable request.

## Declaration of interests

No conflicts of interest relevant to this submission reported.

## Appendix A Supplementary data

Supplementary Data and Code Link
