# Supplementary material for: The burden of childhood cancers in the South Asian Association for Regional Cooperation (SAARC) Region: a population-based, cross-sectional GLOBOCAN 2022 analysis
Source: Lancet Reg Health Southeast Asia. 2026 Jul 14;52:100815. doi: 10.1016/j.lansea.2026.100815 (PMC13382643; doi:10.1016/j.lansea.2026.100815)
Supplement: Supplementary Data and Code Link [file mmc1.docx]

<https://drive.google.com/drive/folders/1Znvt-13kHyDZA8zv1q3ShKyheHXU5yyc?usp=sharing> (Google Drive link to supplementary data and code file, uploading to Lancet portal uploads all files one by one, which is clogging submission)

SUPPLEMENTARY DATA FILE

The Burden of Childhood Cancers in the South Asian Association for Regional

Cooperation (SAARC) Region: A Population-Based, Cross-Sectional GLOBOCAN 2022

Analysis

Manuscript ID D-26-01358R2 — The Lancet Regional Health – Southeast Asia

Corresponding author: Edward Christopher Dee

This file is provided in fulfilment of the manuscript's Data Sharing

statement: "The country- and sex-specific GLOBOCAN 2022 data extracts

underlying all reported estimates, together with the analytic code, are

provided as a supplementary data file accompanying this submission."

================================================================================

CONTENTS

================================================================================

raw_data/ 20 original GLOBOCAN 2022 (IARC, https://gco.iarc.who.int)

CSV extracts, unmodified from source, underlying every

incidence, mortality, ASR, ASMR, and MIR estimate reported

in the manuscript (Tables 1-3; Figures 1-4).

analytic_code/ Python scripts that load the files in raw_data/ (via a

relative path, so the package runs standalone) and

reproduce the exact published numbers and figures. Each

script is independently runnable: `python3 <script>.py`

(requires pandas, numpy, matplotlib; figure scripts also

require scipy and seaborn).

================================================================================

FILE-BY-FILE MAP: raw_data/ -> where it is used

================================================================================

dataset-inc-both-sexes-age-0-14-in-2022-afghanistan.csv

dataset-inc-both-sexes-age-0-14-in-2022-bangladesh.csv

dataset-inc-both-sexes-age-0-14-in-2022-bhutan.csv

dataset-inc-both-sexes-age-0-14-in-2022-india.csv

dataset-inc-both-sexes-age-0-14-in-2022-maldives.csv

dataset-inc-both-sexes-age-0-14-in-2022-nepal.csv

dataset-inc-both-sexes-age-0-14-in-2022-pakistan.csv

dataset-absolute-numbers-inc-both-sexes-age-0-14-in-2022-sri-lanka.csv

Per-country, both-sexes incidence (all cancer types, ages 0-14, 2022).

Used in: Table 1 (incidence n/%, ASR), Table 2, Figure 2 (country x

cancer-type composition/heatmap).

dataset-mort-both-sexes-age-0-14-in-2022-afghanistan.csv

dataset-mort-both-sexes-age-0-14-in-2022-bhutan.csv

dataset-mort-both-sexes-age-0-14-in-2022-india.csv

dataset-mort-both-sexes-age-0-14-in-2022-nepal.csv

dataset-mort-both-sexes-age-0-14-in-2022-pakistan.csv

dataset-mort-both-sexes-age-0-14-in-2022-sri-lanka.csv

dataset-absolute-numbers-mort-both-sexes-age-0-14-in-2022-bangladesh.csv

Per-country, both-sexes mortality (all cancer types, ages 0-14, 2022).

Used in: Table 1 (deaths n/%, ASMR, MIR), Table 3 (cancer-type death

distribution by country). No mortality file exists for Maldives or

sex-stratified per-country mortality in GLOBOCAN 2022 -- these are

reported as "··" (not available) in Table 1/Figure 1C, consistent

with the manuscript text.

dataset-absolute-numbers-inc-and-mort-both-sexes-age-0-14-in-2022-afghanistan-bangladesh-bhutan-sri-.csv

dataset-absolute-numbers-inc-and-mort-males-and-females-age-0-14-in-2022-afghanistan-bangladesh-bhut.csv

SAARC-wide incidence and mortality by cancer type, both sexes and

sex-stratified. Used in: Table 2, Figure 4 (incidence/mortality/MIR

by cancer type, including sex-stratified MIR panel).

dataset-absolute-numbers-inc-males-and-females-age-0-14-in-2022-all-cancers.csv

Per-country male and female incidence, all cancers. Used in: Figure 1

Panel B (male vs female ASR by country).

dataset-absolute-numbers-inc-both-sexes-age-0-14-in-2022-all-cancers.csv

SAARC-wide, both-sexes incidence summary (all cancers). Used in

cross-checks of regional incidence totals reported in Results.

dataset-absolute-numbers-inc-both-sexes-age-0-14-in-2022-continents.csv

Continent-level GLOBOCAN incidence extract, included as supplied in

the original data package for completeness and transparency. It is

not directly cited in any specific reported estimate in this

manuscript (no continent-level comparison figures appear in the

main text or figures).

EXCLUDED FROM THIS PACKAGE (not used in this 2022 cross-sectional analysis):

dataset-absolute-numbers-mort-females-age-0-14-in-2045-afghanistan-bangladesh-bhutan-sri-lanka-india.csv

dataset-absolute-numbers-mort-males-age-0-14-in-2045-afghanistan-bangladesh-bhutan-sri-lanka-india-m.csv

(GLOBOCAN 2045 future-projection extracts -- the manuscript reports

only observed 2022 estimates, not projections.)

================================================================================

ANALYTIC CODE

================================================================================

table1_incidence_mortality_by_country.py

Computes Table 1 (incidence, mortality, ASR, ASMR, MIR by country).

Reproduces SAARC-wide overall MIR = 0.469 reported in the manuscript.

table2_leading_cancer_types_saarc.py

Computes Table 2 (leading cancer types by incidence/mortality, SAARC-

wide). Reproduces MIR = 0.469 (total) and per-type MIRs reported in

the manuscript.

table3_leading_cancer_types_by_country.py

Computes Table 3 (country-specific distribution of leading cancer

types by mortality). Bhutan and Maldives excluded, consistent with

the published table footnote (insufficient case numbers).

figure1_panelsAB_incidence_ASR.py

Reproduces Figure 1 Panels A (absolute incidence by country) and B

(male vs female ASR by country). Figure 1 Panel C (ASMR by sex) is

not regenerated by code: GLOBOCAN 2022 does not provide per-country

sex-stratified mortality data (only both-sexes-combined mortality

files exist for each country, as listed above), so Panel C cannot be

recomputed from any underlying CSV. The published Panel C values

(region-wide male ASMR 4.07 vs female ASMR 2.86 per 100,000) are

given in the manuscript text as SAARC-wide composites, not derived

per-country in this code.

figure2_composition_heatmap.py

Reproduces Figure 2 (cancer-type composition by country and chi-

square standardized-residual heatmap). Reproduces chi2 = 2100.57,

df = 189 (full omnibus test) and chi2 = 1269.08, df = 25 (6x6 subset

shown in the heatmap), matching the manuscript exactly.

figure3_mir_by_country.py

Reproduces Figure 3 (MIR by country, with SAARC-wide male/female MIR

reference lines). Reproduces chi2 = 147.51, df = 5, and SAARC MIR

values (overall 0.469, male 0.474, female 0.463).

figure4_mir_by_cancer_type.py

Reproduces Figure 4 (incidence, mortality, and sex-stratified MIR by

cancer type). Reproduces per-type MIR values reported in the

manuscript (e.g., Leukaemia 0.468, Brain/CNS 0.550, Non-Hodgkin

lymphoma 0.478, Hodgkin lymphoma 0.462, Kidney 0.410).

================================================================================

SOURCE

================================================================================

All data: International Agency for Research on Cancer, GLOBOCAN 2022.

https://gco.iarc.who.int

For questions about this data package, contact the corresponding author,

Edward Christopher Dee, or the first author, Tara Pattilachan Menon, on

reasonable request, per the manuscript's Data Sharing statement.
