# Supplementary material for: The burden of childhood cancers in the South Asian Association for Regional Cooperation (SAARC) Region: a population-based, cross-sectional GLOBOCAN 2022 analysis
Source: Lancet Reg Health Southeast Asia. 2026 Jul 14;52:100815. doi: 10.1016/j.lansea.2026.100815 (PMC13382643; doi:10.1016/j.lansea.2026.100815)
Supplement: Supplementary File [file mmc2.docx]

**Supplementary Table 1:** Cancer incidence data sources for South Asian countries in GLOBOCAN

| **Country** | **Cancer incidence data sources** |
| --- | --- |
| Afghanistan | Mean of estimated rates from Tajikistan, Pakistan and Uzbekistan applied to 2022 population. |
| Bangladesh | Mean of the rates from India: Meghalaya, Tripura and Cachar (2010-2016) applied to 2022 population. |
| Bhutan | Mean of the rates from India: Kamrup Urban District, Dibrugarh District, Sikkim State and Mizoram State (2007-2016) applied to 2022 population. |
| India | Weighted average of the rates from Mumbai, Chennai, Nagpur, Poona, Barshi, Trivandrum, Dindigul Ambilikkai, Aurangabad, Kamrup Urban District, Dibrugarh District, Sikkim State, Mizoram State, Wardha, Kollam, Tripura, Ratnagiri, Meghalaya, Cachar, Nagaland and Sindhudurg (2010-2016) projected to 2022 and applied to 2022 population. |
| Maldives | Mean of estimated rates from Indonesia applied to 2022 population. |
| Nepal | Mean of the rates from India: Sikkim State, Pasighat, Kamrup Urban District and Dibrugarh District (2013-2016) applied to 2022 population. |
| Pakistan | Rates from Punjab (2008-2017) projected to 2022 and applied to 2022 population. |
| Sri Lanka | Rates from Colombo district (2013-2017) applied to 2022 population. |

** From IARC's Global Cancer Observatory Cancer Today* <https://gco.iarc.who.int/today/en/data-sources-methods-by-country-detailed>
